# Pancreatic and splenic tuberculosis mimicking a pancreatic cancer with splenic metastases in an immunocompetent adolescent: a case report

**DOI:** 10.1093/jscr/rjac077

**Published:** 2022-03-22

**Authors:** Joel Gabin Konlack Mekontso, Guy Loic Nguefang Tchoukeu, Fabrice Leo Tamhouo Nwabo, Ulrich Igor Mbessoh Kengne, Michel Arsène Nana Gwabap, Yves Alain Notue, Boniface Moifo, Linda Ngueffo Sando, Angèle Clarisse Kabeyene Okono

**Affiliations:** 1 Ministry of Public Health, Djeleng Subdivisional Hospital, Bafoussam, Cameroon; 2 Ministry of Public Health, Mengueme Subdivisional Hospital, Mengueme, Cameroon; 3 Department of Surgery, Faculty of Medicine and Biomedical Sciences, University of Yaoundé I, Yaoundé, Cameroon; 4 Ministry of Public Health, Surgery Unit, Mifi District Hospital, Bafoussam, Cameroon; 5 Ministry of Public Health, Surgery Unit, Eseka District Hospital, Eseka, Cameroon; 6 Ministry of Public Health, Department of Pathology, Gynaeco-Obstetric and Pediatric Hospital, Yaoundé, Cameroon; 7 Department of Radiology, Radiotherapy and Medical Imaging, Faculty of Medicine and Biomedical Sciences, University of Yaoundé I, Yaoundé, Cameroon; 8 Department of Pathology, Faculty of Medicine and Biomedical Sciences, University of Yaoundé I, Yaoundé, Cameroon

**Keywords:** splenic tuberculosis, pancreatic tuberculosis, pancreatic cancer, Cameroon

## Abstract

We present a case of pancreatic and splenic tuberculosis (TB) in a 15-year-old human immunodeficiency virus-negative patient who was initially misdiagnosed as suffering from a pancreatic carcinoma with splenic metastases. Pancreatic and splenic TB are extremely rare in young immunocompetent patients, with a nonspecific clinical presentation, making the diagnosis elusive.

## INTRODUCTION

Despite majors advances, tuberculosis (TB) is still common in developing countries [1, 2]. Children under 5 years of age [3] and human immunodeficiency virus (HIV)-infected/immunosuppressed patients are mostly affected [2, 4–6]. Only 12% of all extrapulmonary TB involve the abdomen [7]. Pancreatic and splenic TB are extremely rare in HIV-negative patients [4, 7, 8]. A single case has been reported in Cameroon [9]. The most common presenting symptoms are splenomegaly, fever, fatigue and weight loss; therefore, it is likely to be misdiagnosed as a malignancy, especially when there is no prior history of pulmonary TB [6]. We report a rare case of pancreatic and splenic TB mimicking pancreatic carcinoma with splenic metastases in a 15-year-old immunocompetent patient.

## CASE PRESENTATION

**Figure 1:**
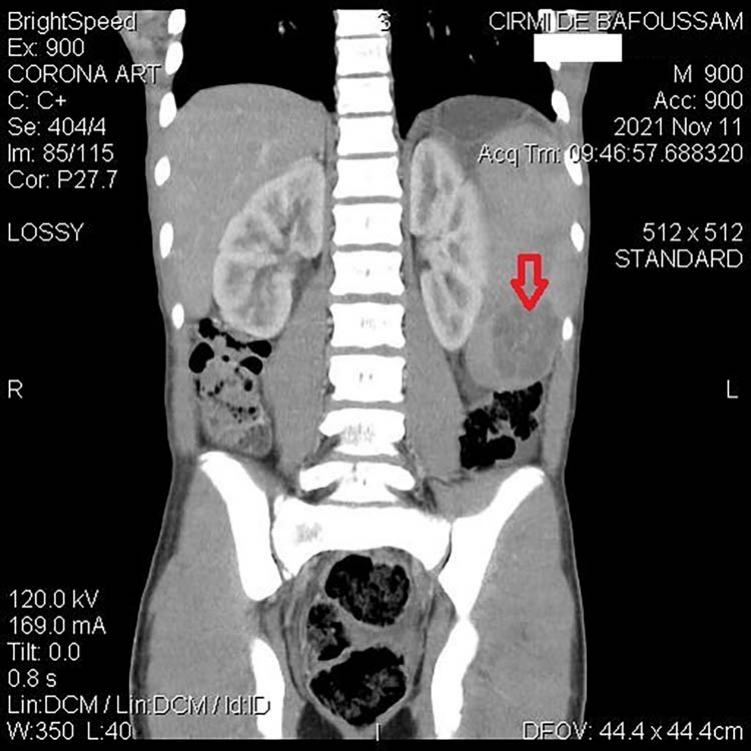
Abdominal CT demonstrating a contrast-enhanced hypodense mass in the spleen.

**Figure 2:**
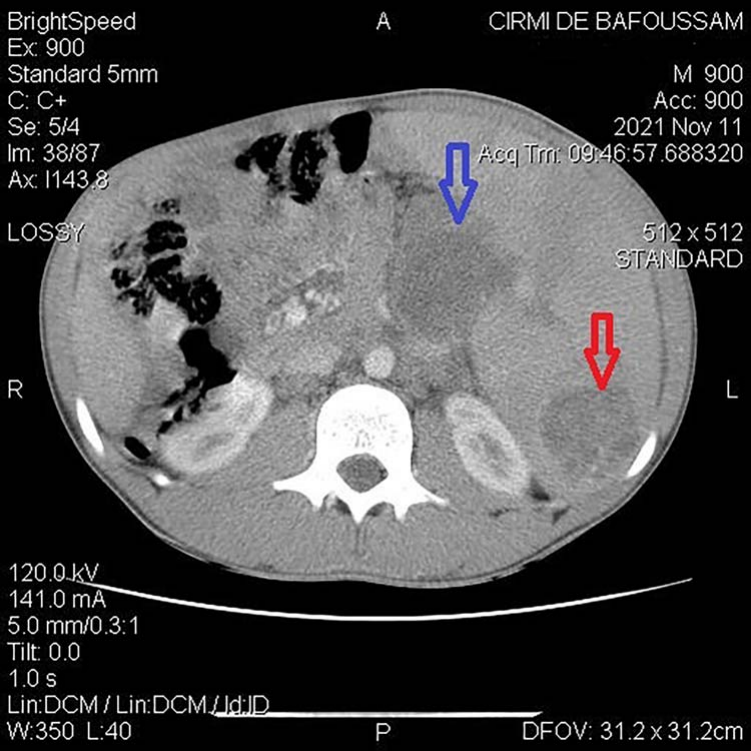
Abdominal CT demonstrating a cystic mass in tail of the pancreas (top arrow) and the tip of the spleen (bottom arrow).

**Figure 3:**
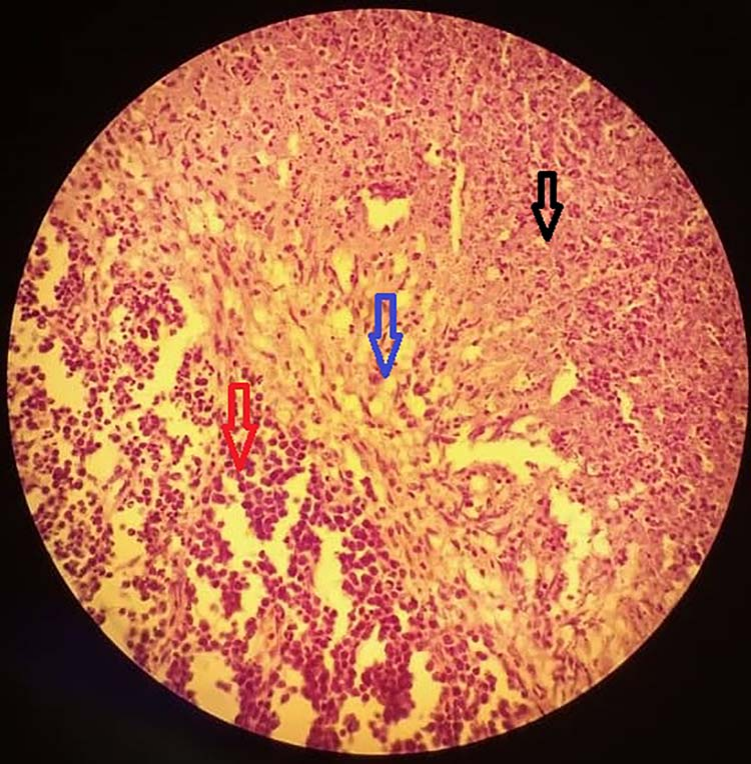
A section of the biopsy specimen showing a granuloma made up of lymphoid cells (left arrow), Langerhan’s giant cells and epithelioid cells (middle arrow) and caseous necrosis (right arrow); (hematoxylin and eosin stained technique, middle-multiplications).

A 15-year-old patient presented with a 3-week history of left upper quadrant abdominal pain with no other symptoms. He had no prior history of pulmonary TB. His vital signs were as follows: temperature = 37°C, blood pressure = 120/78 mmHg, pulse = 84/min, respiratory rate = 15/min, SpO_2_ = 96% on room air; and physical examination only revealed a mild tenderness in the left upper quadrant with the tip of the spleen expanding to the umbilicus. An abdominal ultrasound (US) followed by a computed tomography (CT) scan revealed a heterogeneous splenomegaly with multiple cystic masses in the spleen and the tail of the pancreas (Figs 1 and 2). Laboratory exams showed a white cell count of 2600/mm^3^, hemoglobin of 11.4 g/dl, C-reactive protein (CRP) of 34 mg/l, hemoglobin electrophoresis AA and negative malaria and HIV tests. We suspected a primary or secondary malignancy of the spleen, like a lymphoma or a metastatic pancreatic carcinoma. The other diagnoses were tropical splenomegaly and splenic infarct. We performed a distal pancreatectomy with splenectomy, and histopathological analysis revealed caseating granulomas of the spleen (Fig. 3) with no malignancy suggesting TB. Further, a Ziehl-Neelsen stain of the specimen showed acid fast bacilli (AFB). The patient received anti-TB drugs, had an uneventful post-operative course and was discharged on post-operative day 7. On the first month follow-up visit, he reported marked improvement of his symptoms.

## DISCUSSION

Ninety percent of TB locate primarily in the lungs [10]. In 6–38% of cases, abdominal and active pulmonary TB coexist [4]. Coley described the first case of splenic TB in 1846 [2, 8]. In 1912, Winternitz classified it as primary or secondary. The secondary form occurs as part of miliary TB, especially in immunocompromised patients and is not rare [4, 7, 8, 11]. The primary form is limited to the spleen, the original focus having healed [8, 11] as in our case.

Splenic TB is more common in men [2] and has been reported in immunocompetent patients from 19 to 65 years of age [2, 4, 6, 8, 9]. To the best of our knowledge, our patient is the youngest case ever reported. This case is relevant because pancreatic and splenic TB are extremely rare in young HIV-negative patients. Moreover, fever with splenomegaly in a pediatric patient in Cameroon will primarily be diagnosed as malaria by most physicians. Issues like overcrowding and poor housing conditions, low access to health services, poor nutrition, low income or lack of sanitation could favor the occurrence of TB in developing countries [9].

The clinical presentation is nonspecific and misleading. It ranges from abdominal pain with splenomegaly, as in our case, to fever of unknown origin, pallor, fatigue and weight loss [2, 9]. Early consultation could explain why our patient lacked other symptoms.

Laboratory results typically reveal leukocytosis, anemia, thrombocytopenia and elevated CRP. These findings are not very useful for diagnosis as they can be seen in any chronic inflammatory process [2, 9].

Imaging is helpful. US examination is simple, non-invasive and typically reveals hypoechoic micro- or macronodules. The CT scan is highly sensitive [2, 9]. However, none of them can suggest the nature of the lesions as many situations can present alike, such as lymphomas, metastatic cancers, echinococcal cysts, hemangiomas or fungal splenic abscesses [10].

Histopathological examination is the gold standard in confirming the diagnosis. Samples are obtained by US or CT-guided biopsies [2, 6, 9]. When the needle biopsy is risky (perforation of a viscus and rupture a large cyst) or when TB is not highly suspected enough, as it was the case with our patient, exploratory laparotomy with splenectomy may be both diagnostic and therapeutic [6, 9]. Laparoscopy can be used when available and has helped avoid morbid surgeries [6, 9, 10]. The specimens are examined both macroscopically and microscopically, with typical manifestation being caseating granulomas with epithelioid cells and Langhan’s cells [10] like with our patient. Ziehl-Neelsen stain is also helpful and shows acid fast bacilli suggestive of TB. Although it was not the case with our patient, non-caseating granulomas should raise suspicion of sarcoidosis [9].

The acid fast bacilli can also be detected by the polymerase chain reaction technique. Culture helps to perform drug susceptibility testing [5, 9] but we did not carry out these procedures.

Anti-TB drugs should always be given whether splenectomy has been done or not. The treatment regimen includes four drugs (RHEZ) for 2 months, followed by two drugs (RH) for 4 months [2, 4, 9] and must be adapted in drug-resistant TB.

The post-operative complications are not different from those of other indications of laparotomy. High morbidity and mortality is typically seen in older patients, consulting late, with an altered general state [9]. However, younger patients consulting early and treated in a multidisciplinary setting, as in our case, typically have an uneventful post-operative course.

## CONCLUSION

We presented a rare case of pancreatic and splenic TB in a young immunocompetent adolescent with no prior history of pulmonary TB. The diagnosis is challenging because of the nonspecific clinical presentation. Physicians should always consider TB in patients presenting with cystic pancreatic or splenic masses in TB-endemic regions regardless their HIV status.

## CONFLICT OF INTEREST STATEMENT

None declared.

## FUNDING

This work received no specific grant from any funding agency in the public, commercial or not-for-profit sectors.

## PATIENT CONSENT

Obtained.
